# Vascular Dynamics During Respiratory Arousal From Sleep Assessed by Oximeter‐Based Vascular Stiffness Measures

**DOI:** 10.1111/jsr.70343

**Published:** 2026-04-26

**Authors:** Christian Strassberger, Ding Zou, Jan Hedner, Ludger Grote

**Affiliations:** ^1^ Center for Sleep and Vigilance Disorders, Institute of Medicine, Sahlgrenska Academy University of Gothenburg Gothenburg Sweden; ^2^ Center for Sleep Medicine, Department of Respiratory Medicine Sahlgrenska University Hospital Gothenburg Sweden

**Keywords:** arousal, arterial stiffness, cardiovascular risk, obstructive sleep apnea, photoplethysmography, pulse propagation time

## Abstract

Cortical arousals significantly impact obstructive sleep apnoea (OSA) pathophysiology and cardiovascular consequences, often mediated by sympathetic activation. While pulse oximetry (PPG) provides an accessible reflection of autonomic responses such as changes in pulse rate (PR) and pulse wave amplitude (PWA), the dynamic influence on other vascular indices remains less explored. We investigated dynamic changes in PPG‐derived pulse propagation time (PPT), a metric reflecting modulation in vascular stiffness. It is hypothesised that PPT beat‐to‐beat responses to arousals are specific and independent from other pulse wave parameters. Polysomnography data from 87 patients were analysed (mean age 54 ± 12 years, 26% female, mean BMI 27.7 ± 3.6 kg/m^2^, mean Apnea‐Hypopnea Index 33 ± 23 events/h; total 18,275 respiratory events). The mean percentage change (shortening, vascular stiffening) of the PPT following respiratory events was significantly greater when accompanied by an EEG arousal in both apnoeas (−21.1% vs. −17.9%, *p* < 0.001) and hypopneas (−19.4% vs. −17.0%, *p* < 0.001). In a multivariate logistic mixed‐effect model, ∆PPT emerged as an independent predictor of EEG arousal presence (*p* < 0.001, scaled odds ratio: 1.14; 95% CI: 1.09–1.20), accounting for event type, anthropometrics and changes in PWA, PR, SpO_2_. Dynamic, short‐term changes in PPG‐derived PPT are significantly associated with cortical arousals following respiratory events in OSA patients. This parameter provides independent predictive information beyond established PPG metrics, likely reflecting rapid, arousal‐induced alterations in vascular tone. These findings highlight a novel PPG‐based marker that could quantify the vascular stress in OSA.

Arousals from sleep are a hallmark of obstructive sleep apnoea (OSA). These arousals, while crucial for restoring airway patency, contribute to acute surges in sympathetic nervous system activity and subsequent hemodynamic changes. Intermittent hypoxia and arousals from sleep are associated with an increased risk for cardiovascular consequences, including hypertension and endothelial dysfunction (Javaheri et al. 2024). Detection of arousal from sleep uses polysomnography (PSG) with electroencephalography (EEG), a resource‐demanding procedure. In contrast, autonomic responses to cortical arousals can be monitored using photoplethysmography (PPG) in finger pulse oximetry, typically assessed through changes in pulse rate (PR) and pulse wave amplitude (PWA), reflecting cardiac and peripheral blood flow responses (Grote et al. 2003; Catcheside et al. 2002). While the sympathetic activation causes vasoconstriction seen in the PWA, related vascular stiffening and surges in blood pressure will increase the speed of the pulse wave and alter its reflection wave. The dynamic impact of OSA events on these changes in arterial stiffness on a beat‐to‐beat basis is less well understood.

Pulse transit time (PTT), defined as the time delay from the ECG R‐wave to the foot of the PPG wave, is a non‐invasive measure that reflects the interplay between blood pressure and arterial stiffness (Pollak and Obrist 1983). This physiology can also be estimated from the PPG waveform alone, utilising the pulse propagation time (PPT) (Svedmyr et al. 2016). This metric reflects the time it takes for the arterial pulse wave to travel from the heart to a peripheral site and back as a reflection. Both the PTT and the PPT are fundamentally influenced by the stiffness of the arterial walls. Previous studies from our group have used an overnight average of PPT, linking this static marker of arterial stiffness to hypertension and long‐term cardiovascular risk (Svedmyr et al. 2016; Sommermeyer et al. 2016). To further develop this principle, we developed a novel method to assess PPT dynamically by decomposing each individual PPG waveform into two curves, hypothesised to represent the initial cardiac ejection wave and the summed reflected waves returning from peripheral sites (Figure 1a) (Nagasawa et al. 2022). The time interval between the peaks of these two components provides a beat‐to‐beat measure of PPT, allowing for a nuanced assessment of rapid changes in vascular tone. In this work, we investigate the association between the PPT fluctuations and the primary determinants of sympathetic activation in OSA: respiratory‐event‐induced cortical arousals and intermittent hypoxia.

**FIGURE 1:**
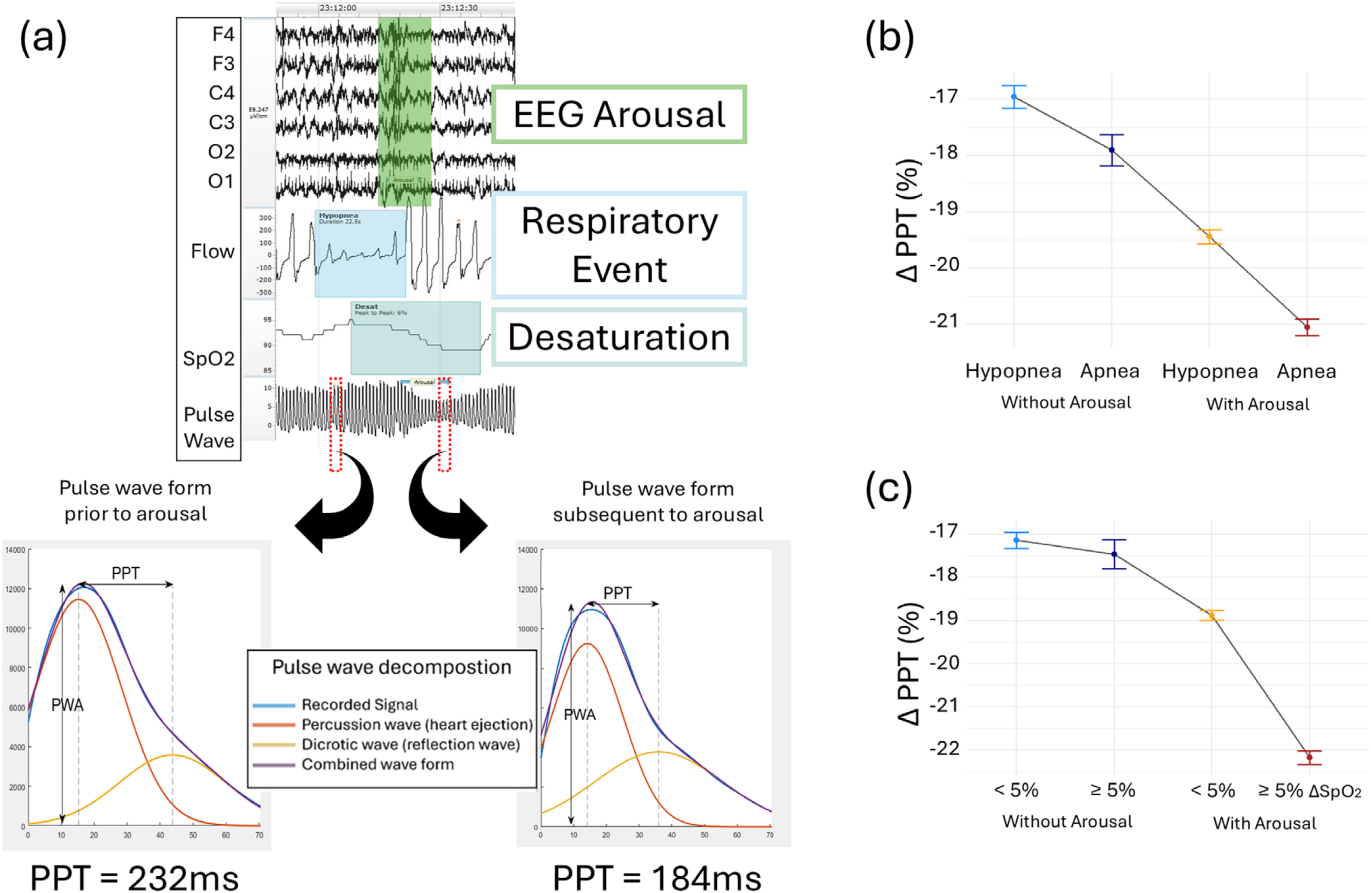
Method and primary results. (a) Example of a respiratory arousal from sleep following a hypopnea. The upper panels illustrate the signals from polysomnography. The PPG‐based pulse wave (blue) is decomposed into an ejection wave (red) and the summed reflection waves (yellow). PPT is the time between their peaks. (b) Stepwise increase in mean PPT shortening (±SE), corresponding to increased vascular stiffness, across event types, from hypopnea without arousal (mildest) to apnea with arousal (strongest). (c) Mean PPT shortening (±SE) stratified by arousal presence and SpO_2_ drop severity, showing an additive effect of hypoxia and arousal. PPG—photoplethysmography, PPT—pulse propagation time, SE—standard error; SpO_2_—oxygen saturation.

We performed a hypothesis‐driven analysis of gold‐standard, ambulatory PSG data from a sleep clinic cohort of 87 patients (mean age 54 ± 12 years, 26% female, mean BMI 27.7 ± 3.6 kg/m^2^, mean Apnea‐Hypopnea Index 33 ± 23 events/h). The study was performed according to the Helsinki Declaration for clinical trials, and the protocol was approved by the local Ethics Committee (DNR: 2019‐05096). The PSG recordings were manually scored according to the AASM 2017 criteria (Berry et al. 2017). A total of 18,275 respiratory events (apnoeas/hypopneas) were identified. For each event, we computed the percentage change in beat‐to‐beat measures of PPT, PWA and PR during a patient‐specific search window following event termination (Azarbarzin et al. 2021). Additionally, the associated drop in blood oxygen saturation (SpO_2_) and the presence of manually scored cortical arousals were recorded for each event. Statistical analyses were performed using R, version 4.3.3 (R Foundation for Statistical Computing, Vienna, Austria). Linear and logistic mixed‐effects models were used to account for repeated measures within subjects.

Our analysis revealed a clear, stepwise physiological response reflected in PPT. The magnitude of PPT shortening, indicating an acute increase in vascular stiffness, was progressively greater across respiratory event types and coexisting central nervous system response (Figure 1b, Table 1). The mildest response was seen for hypopneas without arousals (mean change, −17.0%), followed by apnoeas without arousals (−17.9%), hypopneas with arousals (−19.4%) and finally, apnoeas with arousals (−21.1%), resulting in the most pronounced PPT shortening (ANOVA: *p* < 0.001). This demonstrates that both the type of respiratory disturbance and the presence of cortical arousal have additive effects on the vascular response.

**TABLE 1:** Data Figure 1b,c.

Event type	*n*	Mean ∆PPT %	SE ∆PPT %
Hypopnea without Ar	2432	−16.97	0.20
Apnoea without Ar	1338	−17.91	0.28
Hypopnea with Ar	8219	−19.44	0.12
Apnoea with Ar	5978	−21.05	0.15
Less than 5% Desat without Ar	3056	−17.14	0.18
More than 5% Desat without Ar	672	−17.46	0.33
Less than 5% Desat with Ar	9126	−18.88	0.12
More than 5% Desat with Ar	4767	−22.17	0.16

*Note:* Summary of the percentage change in PPT following respiratory events of different types. Dose response relations are observed for events of increasing severity, that is, hypopnea versus apnoea, or events with and without a significant desaturation of ≥ 5%. Responses were more pronounced in the presence of arousals.

Abbreviations: Ar—arousal; Desat—desaturation; ∆PPT%—percentage change in pulse propagation time, SE—standard error.

Similarly, we observed a dose–response relationship between the severity of hypoxia, the presence of arousals and the change in PPT (Figure 1c, Table 1). Events with both a significant desaturation (≥ 5%) and an EEG‐defined arousal resulted in the most notable increase in vascular stiffness (mean change, −22.2%), a substantially greater response than that seen for events with mild desaturation without arousals (−17.1%, ANOVA: *p* < 0.001).

To establish an additional independent relationship between the shortening of PPT and the presence of arousal, we performed additional sequential modelling (Table 2): First, a logistic mixed‐effects model was built to predict the presence of arousal, using changes in PWA, PR and SpO_2_, along with the type of event. This base model performed well, with all variables being significant predictors, independent of the confounders age, gender and BMI. Adding PPT to this model resulted in a modest, though significant and independent effect size in the context of arousal prediction (odds ratio = 1.14, *p* < 0.001), even after accounting for all other predictors. A likelihood ratio test confirmed a statistically superior fit over the base model (*p* < 0.001). Multicollinearity diagnostics showed that PPT was non‐redundant with other metrics (variance inflation factor [VIF] = 1.03, Table 3). This confirms that PPT provides additional information about the arousal response, not captured by other PPG metrics. Further comparison of model predictions revealed that false negative events (i.e., cortical arousals without strong autonomic response) are characterised by a higher fraction of hypopneas, less severe desaturations and overall shorter duration (Table 4).

**TABLE 2:** Full model to predict presence of arousal in respiratory events.

	Estimate	Std. error	*z* value	*p*
Intercept	1.595	0.1058	15.07	< 0.001
∆PWA (%, standardised)	−0.604	0.0240	−25.15	< 0.001
∆PR (%, standardised)	0.533	0.0361	14.76	< 0.001
∆SpO_2_ (%, standardised)	0.229	0.0351	6.52	< 0.001
Type: Hypopnea	0.146	0.0514	2.84	0.004
∆PPT (%, standardised)	−0.134	0.0251	−5.34	< 0.001

*Note:* Mixed effects model, controlled for subject specific intercept and covariates age, gender and body mass index. PPT had a modest, but statistically significant effect size on the arousal prediction in presence of the established PPG‐based markers of arousals PWA and PR.

Abbreviations: PPT—pulse propagation time; PR—pulse rate; PWA—pulse wave amplitude; SpO_2_—oxygen saturation.

**TABLE 3:** Variance inflation factor.

	VIF	95% CI
∆PWA (%, standardised)	1.10	[1.08–1.12]
∆PR (%, standardised)	1.13	[1.11–1.15]
∆SpO_2_ (%, standardised)	1.19	[1.17–1.21]
Type: Hypopnea	1.15	[1.13–1.17]
∆PPT (%, standardised)	1.03	[1.02–1.05]

*Note:* Multicollinearity diagnostics based on the variance inflation factor (VIF). The low VIF confirms the absence of multicollinearity and therefore the non‐redundant effects of the PPG‐derived metrics.

Abbreviations: CI—confidence interval; PPT—pulse propagation time; PR—pulse rate; PWA—pulse wave amplitude; SpO_2_—oxygen saturation.

**TABLE 4:** Characterisation of events by model prediction.

	False negative	True negative	True positive	False positive
Model prediction	Respiratory event without predicted arousal	Respiratory event without predicted arousal	Respiratory event with predicted arousal	Respiratory event with predicted arousal
Manual scoring	Scored arousal	No arousal	Scored arousal	No arousal
Count	2400	2117	11,472	1605
Type: Apnoea (%)	31.8	33.4	44.3	38.4
∆PPT (%, standardised)	−16.5	−15.5	−20.7	−19.4
∆PWA (%, standardised)	−35.4	−34.3	−55.3	−49.6
∆PR (%, standardised)	7.7	6.9	17.5	13
∆SpO_2_(%, standardised)	2.8	3	4.8	3.5
Arousal duration (s)	8.7	—	12.2	—
Resp. event duration (s)	19.1	19.3	25.4	20.9
Desaturation area (% × s)	73.0	79.1	151.8	108.2

*Note:* Comparison of key autonomic and respiratory event characteristics, separated by model prediction and manual scoring. Positive predictions are driven by strong autonomic responses. False negative events are characterised by shorter duration, lesser desaturations and higher fraction of hypopneas.

Abbreviations: PPT—pulse propagation time; PR—pulse rate; PWA—pulse wave amplitude; SpO_2_—oxygen saturation.

While the strong signals of PWA and PR already capture much of the autonomic response to arousals, our findings demonstrate that this novel, decomposition‐derived PPT is a sensitive marker with independent statistical significance over conventional PPG metrics. It provides a unique window into the acute, event‐related changes in vascular tone. This aligns with the known pathophysiology of OSA, where sympathetic surges are known to cause increases in blood pressure and alterations in the blood flow dynamics. The independence of PPT from PWA and PR suggests it captures a distinct dimension of the physiological response, potentially reflecting the specific changes in arterial wall properties occurring after respiratory arousal from sleep. Combining these parameters into a predictive model for the presence of arousals in respiratory events shows a potential for deriving additional information on autonomic variation in OSA.

The strength of our study lies in the comprehensive and detailed analysis and integration of several cardiovascular and autonomic responses to respiratory events with and without manually scored arousal (single scoring expert). Limitations include the inherent challenge of PPG‐based models in identifying arousals without high autonomic stress and the potential bias in the use of a single expert scorer, which cannot reflect between scorer variability. Furthermore, our analyses are focused on arousal scoring in relation to respiratory events, and further studies are needed to expand our findings on non‐respiratory arousal detection using the PPT signal.

In conclusion, the dynamic beat‐to‐beat assessment of PPT offers a more comprehensive view of the vascular impact of arousal responses in OSA than conventional PPG‐derived metrics alone to assist non‐EEG‐based arousal detection. As a simple, non‐invasive assessment, detailed PPG analyses may be further utilised in clinical practice to quantify the vascular stress caused by individual events beyond the routine assessment of OSA severity based on event frequency. This could enhance our understanding of the mechanisms linking OSA to cardiovascular disease and improve the diagnostic value of oximeter‐based home sleep apnoea testing.

## Author Contributions

**Christian Strassberger:** conceptualization, investigation, writing – original draft, methodology, validation, visualization, software, formal analysis, project administration, data curation. **Ding Zou:** conceptualization, investigation, methodology, supervision, review and editing. **Jan Hedner:** funding acquisition, conceptualization, investigation, methodology, supervision, review and editing. **Ludger Grote:** funding acquisition, investigation, conceptualization, writing – review and editing, methodology, project administration, supervision, resources.

## Funding

This work was supported by Desitin Arzneimittel, Hjärt‐Lungfonden (20180585, 20210529, 20210500), and Swedish state under the agreement between the Swedish government and the county councils, the ALF‐agreement (LUA/ALF 966319, LUA/ALF 966283).

## Conflicts of Interest

C.S. and D.Z. declare no conflicts of interest. J.H. reports lecturing activities for SomnoMed, Desitin, and Bayer Pharma; is also a collaborator in the European Union Horizon 2020 and EUROSTAR programs (Sleep Revolution, ‘WatchIT); has received grants from European Union interreg program Sleep Aross Waters; is an advisory committee member for SomnoMed; and is a co‐owner of a licensed patent for sleep apnea treatment. L.G. reports lecturing activities for Resmed, Philips, Astra Zeneca, and Lundbeck as well as grant support for scientific projects from Bayer and the European Union Horizon 2020 and EUROSTAR programs (Sleep Revolution, APNEWAY); is an advisory committee member for Onera; and is a co‐owner of a licensed patent for sleep apnea treatment.

## Data Availability

The data that support the findings of this study are available on request from the corresponding author. The data are not publicly available due to privacy or ethical restrictions.
